# Effects of Ultrasonic Surface Rolling Processing and Subsequent Recovery Treatment on the Wear Resistance of AZ91D Mg Alloy

**DOI:** 10.3390/ma13245705

**Published:** 2020-12-14

**Authors:** Xiaohui Zhao, Kaichao Liu, Desheng Xu, Yu Liu, Chunhua Hu

**Affiliations:** 1 Key Laboratory of Automobile Materials, School of Materials Science and Engineering, Jilin University, 5988 Renmin Street, Changchun 130025, China; zhaoxiaohui@jlu.edu.cn (X.Z.); liukc18@mails.jlu.edu.cn (K.L.); xuds@jlu.edu.cn (D.X.); huchunhua@jlu.edu.cn (C.H.); 2 School of Mechanical and Aerospace Engineering, Jilin University, 5988 Renmin Street, Changchun 130025, China

**Keywords:** AZ91D Mg alloy, ultrasonic surface rolling processing, recovery treatment, surface nanocrystallization, wear resistance

## Abstract

AZ91D Mg alloy was treated by ultrasonic surface rolling processing (USRP) and subsequent recovery treatment at different temperatures. The dry sliding friction test was performed to investigate the effects of USRP and subsequent recovery treatment on the wear resistance of AZ91D Mg alloy by a ball-on-plate tribometer. The microstructure, properties of plastic deformation layer and worn morphology were observed by optical microscope (OM), scanning electron microscope (SEM), transmission electron microscope (TEM), X-ray diffraction (XRD) analysis and microhardness tester. Results illustrate that the grains of AZ91D Mg alloy surface layer are refined to nanocrystallines. The maximum microhardness of the top surface of the USRP sample reaches 102.3 HV. When USRP samples are treated by recovery treatment at 150 °C, 200 °C and 250 °C, the microhardness of the top surface decreases to 90.68 HV, 79.29 HV and 75.06 HV, respectively. The friction coefficient (FC) and wear volume loss of the USRP-R-150 sample are the lowest among all the samples. The worn surface morphology of the USRP-R-150 sample is smoother than that of other samples, indicating that the wear resistance of AZ91D Mg alloy treated by USRP and recovery treatment at 150 °C is improved significantly.

## 1. Introduction

Magnesium (Mg) alloys are lightweight alloys with the advantages of low density, high specific strength and stiffness and excellent electrical and thermal conductivity that have been widely used in automotive, electronics, aerospace, military and other fields [1,2]. However, the poor wear resistance of Mg alloys severely limits their applications and causes huge economic losses. In order to enhance the wear resistance of Mg alloys, some effective technologies have been proposed, such as surface coating technology [3], laser cladding [4], ion implantation [5], physical vapor deposition (PVD) [6] and surface nanocrystallization (SNC) [7,8]. Among the above processing methods, SNC is a simple, economical and effective method to improve the wear resistance of the material surface. There are many technologies to produce nanostructure layers on the surface of materials, such as electrodeposition [9,10], chemical vapor deposition [11] and severe plastic deformation (SPD) [12,13]. The nanostructure surface layers of materials can improve their mechanical properties notably, especially the tribological property [14].

In recent decades, SPD has been proposed that can produce a certain depth of nanostructure layer on the surface of materials including surface mechanical attrition treatment (SMAT) [15], shot peening (SP) [16], equal channel angular pressing (ECAP) [17], ultrasonic impact (UI) [18] and ultrasonic surface rolling processing (USRP) [19]. According to the relevant research, SPD can enhance the surface microhardness and wear resistance of materials [20]. Many researchers have found that SPD can effectively produce nanostructure layers on the surfaces of Mg alloys and improve the wear resistance of Mg alloys. Xia et al. [21] studied the wear behavior of AZ31 Mg alloy with a nanostructure layer generated by SMAT. The friction coefficient of the SMAT-treated sample was lower than that of the as-received sample under dry sliding conditions. Liu et al. [22] investigated wear properties modifications of Mg-8Gd-3Y alloy treated by SP. When the peening intensity reached 0.46 mmN, nanocrystallines were obtained on the surface layer of the alloy. The average wear volumes of the peened samples were lower than those of the original samples. Different from the above conclusions, Liu et al. [23] found that GW63K Mg alloy with nanostructure surface layers produced by SMAT exhibited worse wear resistance than MC Mg alloy. This phenomenon is due to the low ductility and low toughness caused by the nanostructure layer. Heat treatment is an effective method to improve the toughness of metals. Farshidi et al. [24] investigated the evolution of microstructure and mechanical properties of 6060 Al alloy after SPD and subsequent annealing. After an annealing treatment, the finer grains obtained by SPD expanded, and the strength and ductility of Al samples were also improved. Mao et al. [25] found that the mechanical properties of the SPD layer for Cu-Ni alloy obtained after a recovery treatment were better than those of the alloy that experienced SPD without a subsequent recovery treatment. Li et al. [26] studied the influence of a recovery treatment on the friction behavior of the SPD surface for AZ31 Mg alloy. The results showed that the recovery treatment can improve the wear resistance of the alloy after hammering. The above research shows that heat treatment plays an important role in improving the properties of alloys. However, it is worth noting that most of the previous studies mainly focused on the influence of a single heat treatment temperature on the properties of the materials. Few studies were carried out to investigate the effect of gradient temperatures on the properties of Mg alloy with an SPD layer, especially the wear resistance. Therefore, the effect of gradient heat treatment temperatures on the microstructure and wear resistance of Mg alloy with an SPD layer was studied in this research, which was of great benefit to expand the application of Mg alloy.

In this work, AZ91D Mg alloy was treated by USRP and a subsequent recovery treatment at different temperatures. The microstructure, work-hardening effect and grain refinement mechanism after USRP were investigated. The effect of the recovery treatment at different temperatures on the microstructure and wear resistance of AZ91D Mg alloy after USRP was comparatively analyzed. By comparing the friction and wear characteristics of the samples, the wear mechanism was revealed.

## 2. Materials and Methods

The base material used in this study was AZ91D Mg alloy, and its chemical composition is listed in Table 1. Commercial AZ91D Mg alloys were cut into plate samples measuring 100 mm × 100 mm × 10 mm. The samples were ground with 600, 800 and 1000 grit SiC paper before USRP. Then the samples were cleaned ultrasonically with acetone for 5 min and, finally, dried by flowing cold air.

**Figure 1:**
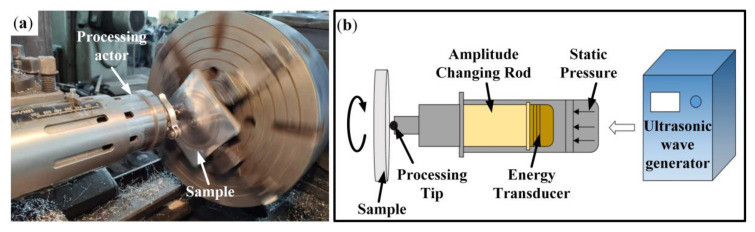
Ultrasonic surface rolling processing (USRP): (**a**) the actual manufacturing process; (**b**) the schematic diagram of USRP.

The surfaces of samples after pre-treatment were treated by USRP equipment (HJ-III, Tianjin University, Tianjin, China) assembled on the machine tool, as shown in Figure 1. Table 2 shows the parameters of USRP used in this work. Five samples were treated by USRP, and the samples with better surface conditions were selected for subsequent experiments. The sample treated by USRP was called the “USRP sample”. The surface roughness of the original sample and the USRP sample was tested by a confocal laser scanning microscope (OLS3000, Olympus, Tokyo, Japan). Before testing, the surface of the original sample was polished with 600, 800 and 1000 grit SiC paper in turn, while the surface of the USRP sample did not need any treatment. Three areas measuring 600 μm × 400 μm were randomly selected to test the surface roughness of each sample, and the average value was taken as the final surface roughness of the sample. Phase structures of the samples before and after USRP were analyzed by X-ray diffraction (XRD) analysis (D8 DISCOVER GDDDS, Bruker, Karlsruhe, Germany) with a Cu Kα radiation source (wavelength: 1.54156 Å). The scanning speed was 0.05°/s and the measuring angle ranged from 20° to 90°. A transmission electron microscope (JEM-2100F, JEOL, Tokyo, Japan) was used to observe the microstructure of the USRP sample. The samples for TEM examination were prepared using the following steps. First, the thin sheet samples with a thickness of 500 μm were cut from the surface of the USRP sample by wire cutting. Then, the thin samples were ground to a thickness of approximately 30 μm and punched into 3 mm-diameter discs. At least three samples were prepared at each depth. Finally, the samples were perforated by an ion milling (RES101, Leica, Wetzlar, Germany) with an ion accelerating voltage of 5 kV at 5 °C.

The recovery treatments of the USRP samples were carried out in the box-type high temperature sintering furnace (KSL-1100X-S-H, Kejing, Hefei, China). According to the previous experimental results, when the recovery temperature was lower than 150 °C, the microstructure and mechanical properties of the samples had no obvious change. Therefore, 150 °C, 200 °C and 250 °C were used for the recovery treatment in this study. The temperature was kept for 30 min and finally furnace cooled. Three samples were used for the recovery treatment at each temperature. During the experiment, samples were protected from oxidation by argon (99.99%). The USRP samples treated by the recovery treatment were called “USRP-R samples”. The microstructures of the original, USRP and USRP-R samples were observed by optical microscope (Scope Axio ZEISS, Jena, Germany). The samples for the optical microscope (OM) examination were ground with 3000 grit SiC paper and first polished with a polishing agent (particle size of 0.5 μm). Then, the samples were corroded in the corrosive solution with 5 g picric acid, 4 mL deionized water, 4 mL acetic acid and 40 mL ethanol for 15 s. Finally, the samples were cleaned ultrasonically with ethanol for 3 min and dried by flowing cold air. Two samples were prepared for each treatment to observe the microstructure. The microhardness of all samples was evaluated by the Vickers microhardness tester (MHVD-1000MP, Jujing, Chizhou, China) with a load of 100 g and loading time of 10 s. On the cross-section of samples, the microhardness was tested at every 10 μm interval by selecting three equivalent points in the horizontal direction from the top surface along the thickness direction, and the average value of the three points was taken as the final microhardness at this depth.

The dry sliding friction and wear test was carried out at room temperature by a ball-on-plate tribometer (MFT-R4000, Huahui, Lanzhou, China). The dimensions of the samples used for the friction and wear tests were 20 mm × 20 mm × 10 mm. The material of the counter grinding ball in this work was GCr15 (diameter = 5 mm, microhardness = 700 HV). All tests were performed under a 10 N normal load and at a frequency of 5 Hz for a sliding time of 30 min. Three samples were used for each treatment to ensure the accuracy of the experiment results. Before the friction and wear test, the sample and GCr15 ball were cleaned ultrasonically with acetone. The morphologies of worn surface and wear particles were investigated by a scanning electron microscope (S-3400, HITACHI, Tokyo, Japan).

## 3. Results and Discussion

### 3.1. Microstructure

**Figure 2:**
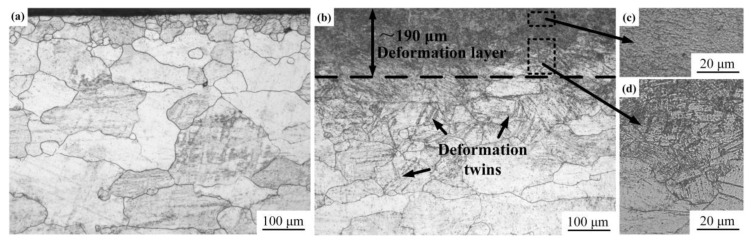
Cross-sectional microstructures of samples: (**a**) Optical microscope (OM) image of original sample; (**b**) OM image of the USRP sample; (**c**) OM image of the deformation layer for the USRP sample; (**d**) OM image of the transition zone of the USRP sample.

Figure 2 shows the cross-sectional microstructures of AZ91D Mg alloy before and after USRP. As shown in Figure 2a, the grains of the original sample are coarse, and there is no obvious difference between the surface layer and the inner part. In contrast, the microstructure of the surface layer is quite different from the inner part after USRP. It can be seen from Figure 2b that severe plastic deformation can be observed in the surface layer of the USRP sample. The thickness of the severe plastic deformation layer is about 190 μm. Compared with the matrix, the grain boundaries of the surface layer can no longer be clearly identified (Figure 2c). Meanwhile, deformation twins are found in Figure 2b (as marked). This is attributed to the plastic flow of the material surface under constant rolling pressure during USRP. At a high strain rate, the slip deformation is restrained, and the stress concentrated near the grain boundaries cannot be released by the slip deformation [27]. Therefore, the tendency of twinning increases and high-density deformation twins are generated after USRP. Figure 2d shows the transition zone between the deformation layer and matrix, and there is no clear boundary between them.

**Figure 3:**
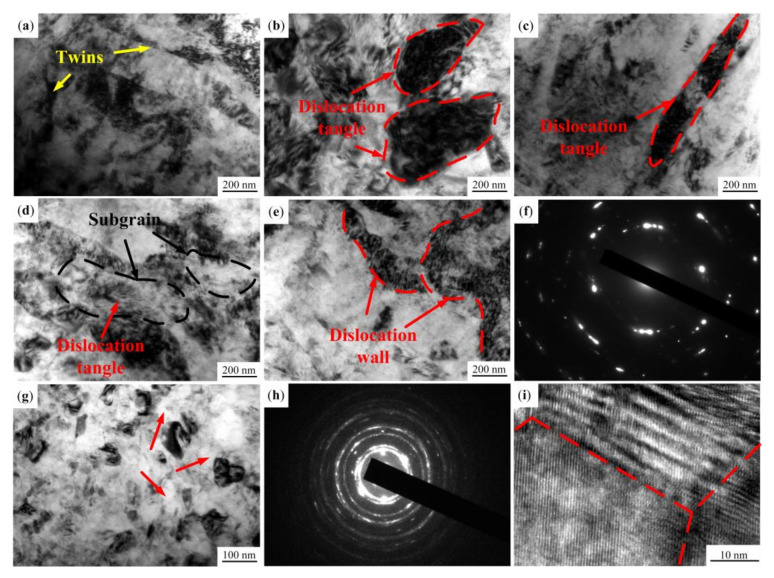
TEM images of the USRP sample: (**a**–**c**) are the microstructure at a depth of 160 μm; (**d**–**f**) are the microstructure and selected area electron diffraction (SAED) pattern at a depth of 80 μm; (**g**–**i**) are the microstructure and SAED pattern at a depth of 10 μm.

Figure 3 shows the TEM bright field images and corresponding selected area electron diffraction (SAED) patterns at a distance of 160 μm, 80 μm and 10 μm from the top surface of the USRP sample. Generally, for polycrystalline materials, the main plastic deformation mechanisms are twinning and dislocation slip [28]. The stacking fault energy (SFE) is an important factor to determine the plastic deformation mechanism of polycrystalline materials. For materials with a high SFE, the plastic deformation mechanism is dislocation slip [29]. The materials with a lower SFE mainly deform in a coordinated manner of twinning and dislocation slip. The SFE of AZ91D Mg alloy is low, so its plastic deformation is mainly coordinated by twinning and dislocation slip.

The TEM bright field images at a depth of 160 μm are shown in Figure 3a–c. The number of slip systems of Mg alloy is very small at room temperature because of the hexagonal close-packed (hcp) crystal structure [30]. As shown in Figure 3a, the deformation twins can be observed in the original grains, indicating that the initial stage of plastic deformation is dominated by twinning. At the same time, under the effect of USRP, the stress on the deformation layer reaches the critical starting stress of a few dislocation slip systems, which start to move and form dislocation. The interaction between dislocations forms dislocation tangles, which divide the original coarse grains into substructures (Figure 3b,c).

Figure 3d–f shows the TEM bright field images and the corresponding SAED pattern at a distance of 80 μm from the top surface. With the decrease of the distance from the top surface, the stress on the deformation layer increases. Therefore, dislocation slip systems are activated and the dislocation slip increases. It can be seen from Figure 3d that dislocation tangles divide the original grains into subgrains. There are high density dislocations in the subgrains, which will further divide the subgrains into finer grains. Meanwhile, when the dislocation movement is hindered, a large number of dislocations pile up to form dislocation walls (as marked in Figure 3e). The SAED pattern shown in Figure 3f is similar to rings, indicating that the grains of the deformation layer have been refined.

Figure 3g–i show the TEM bright field image, the corresponding SAED pattern and the high resolution image at a depth of 10 μm of the USRP sample. As shown in Figure 3g, the grains in the top surface have been transformed into equiaxed grains with random orientation, and the grain sizes range from 50 nm to 70 nm. The SAED pattern consists of continuous diffraction rings, which further confirms that grains of the layer have been refined into nanocrystallines (Figure 3h). Meanwhile, the difference of contrast between the grains illustrates that there is high internal stress on the layer. Figure 3i is a high resolution image at a depth of 10 μm of the sample after USRP. It can be seen that grain orientation is random and characterized by high angle grain boundaries. In addition, twins and dislocations can hardly be found in the top surface, indicating that the most likely type of grain refinement is dynamic recrystallization (DRX). Generally, the recrystallization temperature is about half the *T*_m_, and high strain can decrease the recrystallization temperature. The melting point of Mg alloy is low, so its recrystallization temperature is also low. During USRP, severe plastic deformation occurs on the sample surface at a high strain rate, which converts mechanical work into heat energy. Despite the loss of heat, the temperature can still reach the recrystallization temperature of Mg alloy. The “clean” grains shown in Figure 3g (exhibited by arrowheads) form along the high strain zone, which indicates that dynamic recrystallization has occurred [31].

**Figure 4:**
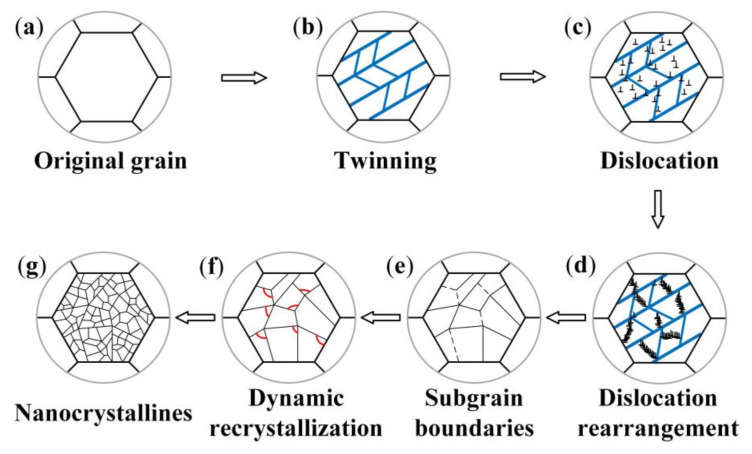
Schematic diagrams of the grain refinement mechanism of AZ91D Mg alloy treated by USRP: (**a**) original grain; (**b**) twinning; (**c**) dislocation; (**d**) dislocation rearrangement; (**e**) subgrain boundaries; (**f**) dynamic recrystallization; (**g**) nanocrystallines.

Figure 4 is a schematic diagram of the process and mechanism of the grain refinement of AZ91D Mg alloy during USRP. As shown in Figure 4b, the severe plastic deformation of AZ91D Mg alloy occurs on the surface, resulting in the formation of deformation twins. With the increase of strain, the number of twins increases. Then, the movement of dislocations is hindered by more and more grain boundaries, leading to dislocation entanglement and pile-up. This results in the increase of grain boundaries, which makes coarse grains transform into fine grains (Figure 4e). In addition, as can be seen from Figure 4f, dynamic recrystallization occurs due to the increase of the strain rate and heat generated during USRP. The dynamic recrystallization results in the formation of nanocrystallines on the surface of AZ91D Mg alloy (Figure 4g). Therefore, the grain refinement mechanism of AZ91D Mg alloy treated by USRP is coordinated by twinning, dislocation slip and dynamic recrystallization.

### 3.2. Surface Roughness

**Figure 5:**
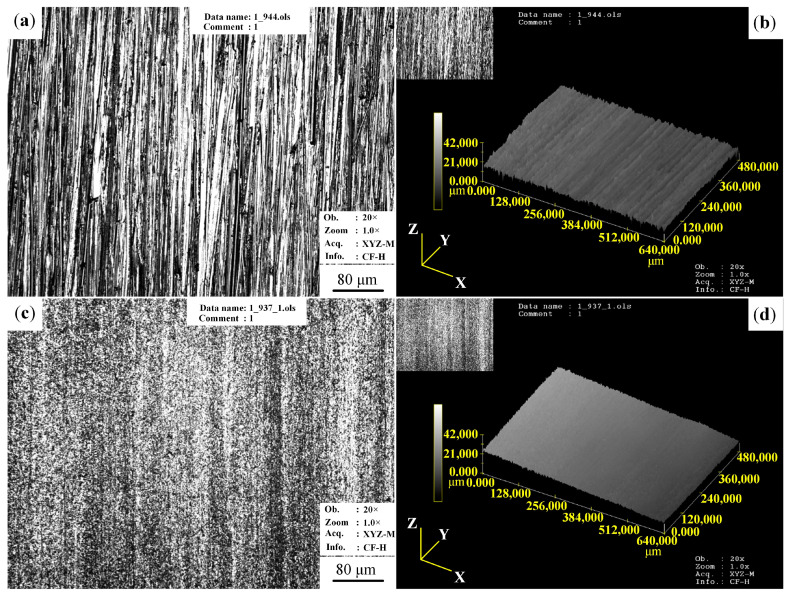
The surface appearance of samples: (**a**) OM image of the original sample; (**b**) three- dimensional appearance of the original sample; (**c**) OM image of the USRP sample; (**d**) three-dimensional appearance of the USRP sample.

Figure 5 shows the 3D surface appearance of the original sample and USRP sample. As can be seen from Figure 5a, a large number of machining marks are distributed on the surface of the original sample after being polished with SiC paper up to 1000 grit. The surface roughness of the original sample reaches 1.283 μm. As shown in Figure 5b, compared with the original sample, the surface of the USRP sample has almost no machining marks, and the uniformly distributed groove marks have disappeared. Therefore, the surface roughness of the sample after USRP is 0.118 μm, which is much lower than that of the original sample.

A similar experimental phenomena is observed in Ye et al. [32]. The reasons for the decrease of surface roughness of the USRP sample are as follows. Firstly, under the high frequency impact of the tool head on the USRP equipment, plastic flow occurs on the surface of Mg alloy, which makes the metal flow from peak to valley. This effect can significantly eliminate the machining defects of the original sample surface, reducing the surface roughness of the sample. Secondly, under the high frequency of ultrasonic, the tool head and the workpiece do not always contact, so the cooling lubricant can enter the gap and reduce the wear between them. As a result, USRP can effectively reduce the surface roughness of AZ91D Mg alloy.

### 3.3. XRD Analysis

**Figure 6:**
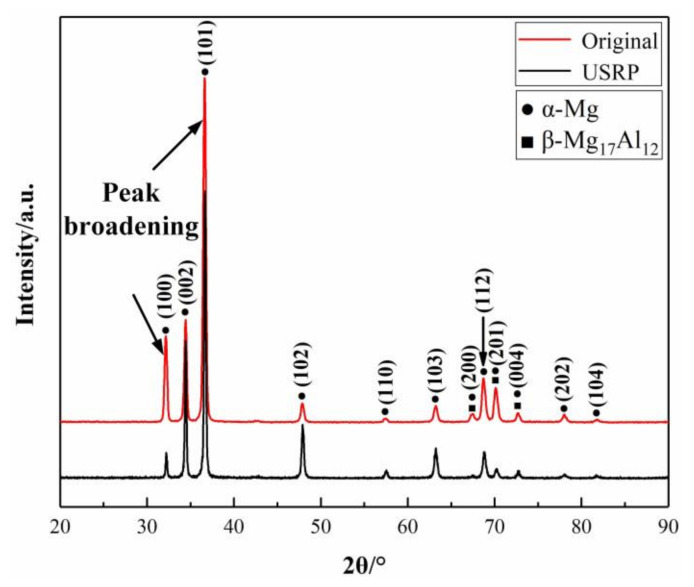
XRD patterns of the original sample and USRP sample.

The XRD patterns of AZ91D Mg alloy for the original sample and USRP sample are shown in Figure 6. It can be seen that there are no diffraction peaks of other phases in the XRD pattern. The original sample and the USRP sample both contain only two phases of α-Mg and β-Mg17Al12, indicating that USRP does not generate new phases in AZ91D Mg alloy. Compared with the original sample, the XRD peaks of the USRP sample are slightly broadened (as marked), which is caused by grain refinement, micro-stress and lattice distortion. The highest diffraction peaks of the original and USRP samples appear at an angle 2θ of 36.6°.

### 3.4. Microhardness

**Figure 7:**
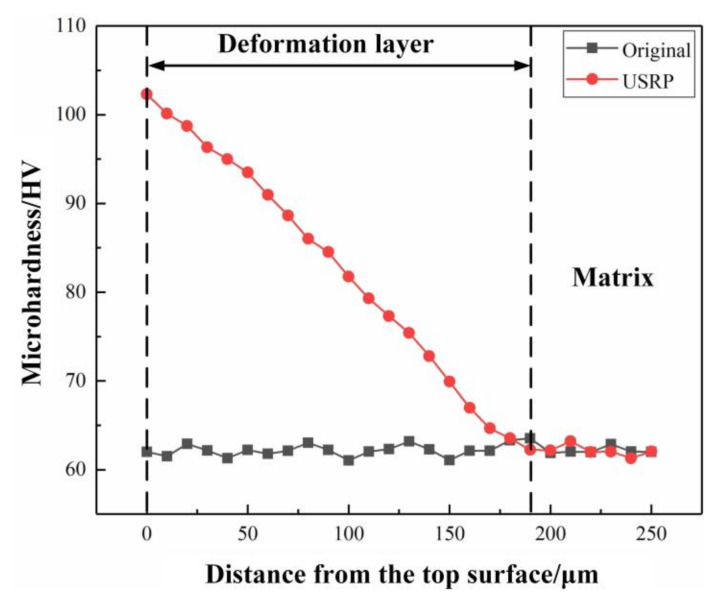
The microhardness distribution with distance from the top surface of the original sample and the USRP sample.

The variations of microhardness with distance from the top surface of the original sample and the USRP sample are shown in Figure 7. It can be seen that the top surface microhardness of the USRP sample reaches 102.3 HV, while the top surface microhardness of the original sample is only 60.02 HV. The highest microhardness of the USRP sample is about 1.7 times larger than that of the original sample, illustrating that the strengthening effect of USRP is remarkable. The reasons for the increase of microhardness are grain refinement, strain strengthening and residual compressive stress caused by USRP. In addition, the microhardness is distributed in gradient along the depth of the cross section of the USRP sample. Meanwhile, the microhardness of the USRP sample is about the same as that of the matrix at a depth of about 190 μm from the top surface. Therefore, the SPD layer thickness of the USRP sample is about 190 μm, which was consistent with the microstructure given in Figure 2b.

The relationship between grain size and microhardness can be explained by the Hall-Petch relationship [33]

*H* = *H*_0_*+ Kd*^−1/2^ (1)

where *H* presents the microhardness of the treated material; *H*_0_ is the microhardness of the base material and *K* is a material constant which depends on the resistance of grain boundaries to dislocation movement; and *d* is the average grain size. If the Hall-Petch relationship holds, then *K* is a positive constant, and there is a linear relationship between the microhardness of materials and the average grain size, so grain refinement is an effective method to improve the microhardness of materials. Some studies use the dislocation plugging model or grain boundary source theory to explain the Hall-Petch relationship [34]. For traditional polycrystalline metals, the free energy at the grain boundaries is high relative to the inner grains, and the grain boundaries can hinder dislocation movement [35]. When dislocations move in polycrystalline metals, the orientations of adjacent grains are different, so the slip band of one grain cannot directly enter the adjacent grains. In order to meet the coordination of grain boundary deformation, multiple slip systems need to act at the same time, which makes it difficult for dislocations to pass through the grain boundary, so as to accumulate at the grain boundary and improve the strength and hardness of the material. Compared with the metals with face-centred cubic (fcc) or body-centered cubic (bcc) crystal structure, Mg alloy is an hcp crystal structure and its *K* is lager, so the strengthening effect after grain refinement is more significant [36].

### 3.5. Microstructure of the USRP-R Sample

**Figure 8:**
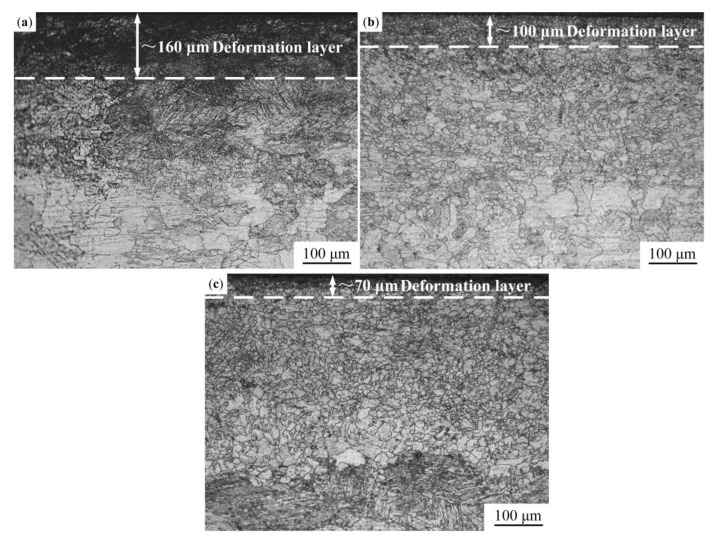
Cross-sectional microstructures of samples: (**a**) USRP-R-150 sample; (**b**) USRP-R-200 sample; (**c**) USRP-R-250 sample.

Figure 8 shows microstructures of the USRP sample after the recovery treatment. It can be seen that the thicknesses of deformation layers of the USRP samples after the recovery treatment decrease. As shown in Figure 8a, when the recovery temperature is 150 °C, the thickness of the deformation layer is 160 μm. In addition, deformation twins in USRP sample eliminate after the recovery treatment. Figure 8b shows the microstructure of the USRP sample after a recovery treatment at 200 °C. It can be found that with the increase of temperature, the grains in the deformation layer further expand, while the size of the coarse original grains in the matrix decreases. The thickness of the deformation layer of the USRP-R-200 sample is approximately 100 μm. The microstructure of the USRP-R-250 sample is similar to that of the USRP-R-200 sample. Compared with the USRP-R-200 sample, the thickness of the deformation layer of the USRP-R-250 sample is smaller, only 70 μm (Figure 8c).

### 3.6. Microhardness of the USRP-R Sample

**Figure 9:**
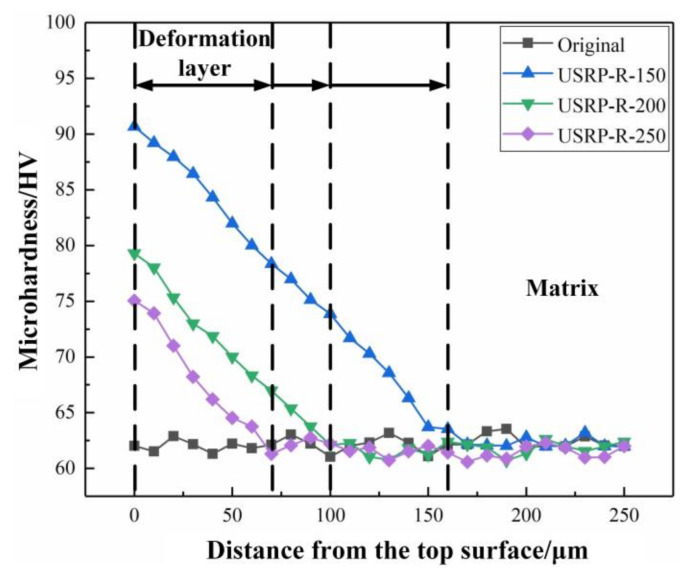
The microhardness distribution with distance from the top surface of the original sample and USRP samples after the recovery treatment at different temperatures.

The microhardness distribution along the depth of the cross section of the original sample and USRP-R samples are shown in Figure 9. The results show that the microhardnesses of the top surfaces of the USRP-R-150 sample, USRP-R-200 sample and USRP-R-250 sample are 90.68 HV, 79.29 HV and 75.06 HV respectively, which are still higher than that of the original sample. Compared with the USRP sample, the microhardnesses of the top surfaces of the USRP-R samples are lower. This is attributed to the grain growth and elimination of residual internal stress after the recovery treatment. At the same time, it can be found that the microhardnesses of the USRP-R samples’ surfaces decreases with the increase of the recovery temperature, which demonstrates that the higher the temperature, the weaker the strengthening effect of USRP.

### 3.7. Friction and Wear Characteristics

**Figure 10:**
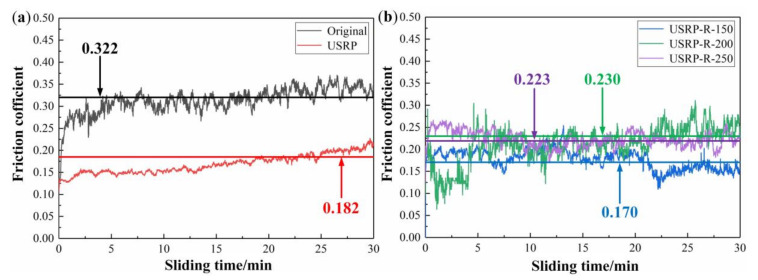
Friction coefficients (FCs) of samples: (**a**) FCs of the original sample and USRP sample; (**b**) FCs of USRP-R samples.

The effects of USRP treatment and subsequent recovery treatment on the friction and wear properties of AZ91D Mg alloy are investigated by the ball-on-plate wear tribometer. Figure 10 shows the variations of the friction coefficient (FC) as a function of the sliding time of the original, USRP and USRP-R samples under an applied normal load of 10 N. It can be seen that there are local fluctuations in the curves, but they tend to be certain values with the increase of time. Meanwhile, the average FCs of the original sample, USRP sample, USRP-R-150 sample, USRP-R-200 sample and USRP-R-250 sample are 0.322, 0.182, 0.170, 0.230 and 0.223, respectively. The FCs of the USRP sample and USRP-R samples are lower than that of the original sample. These results exhibit that USRP can effectively enhance the wear resistance of AZ91D Mg alloy, and further improve the wear resistance of the USRP sample when the recovery temperature is 150 °C.

**Figure 11:**
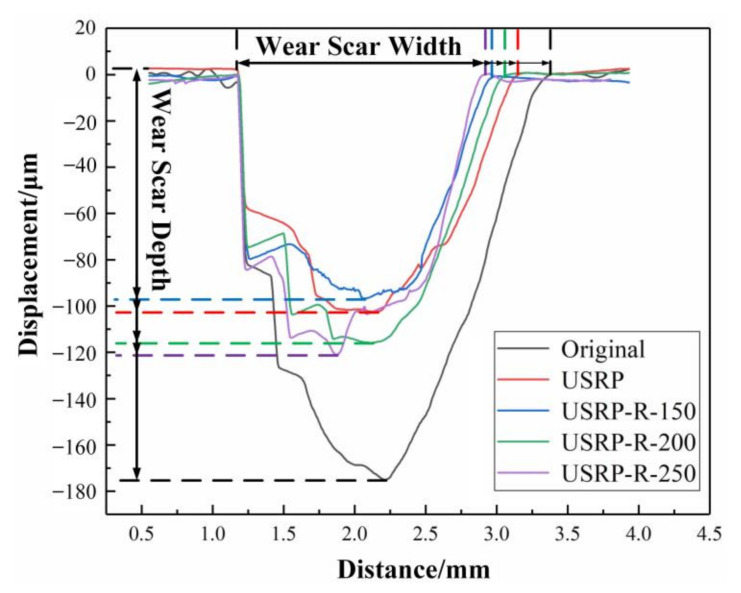
Cross-sectional worn profiles of the original sample, USRP sample and USRP-R samples.

Figure 11 reveals the cross-sectional worn profiles of the original sample, USRP sample and USRP-R samples. As can be seen, the maximum wear depths of samples all appear near the center of wear scars. Then the wear depth is decreased gradually from the center of the wear scar to the side. The wear scar depths of the original, USRP, USRP-R-150, USRP-R-200 and USRP-R-250 samples are about 176 μm, 105 μm, 98 μm, 116 μm and 121 μm, respectively. The wear scar depths of USRP sample and USRP-R samples are shallower than that of the original sample. Among all the samples, the USRP-R-150 sample shows the shallowest wear scar depth.

**Figure 12:**
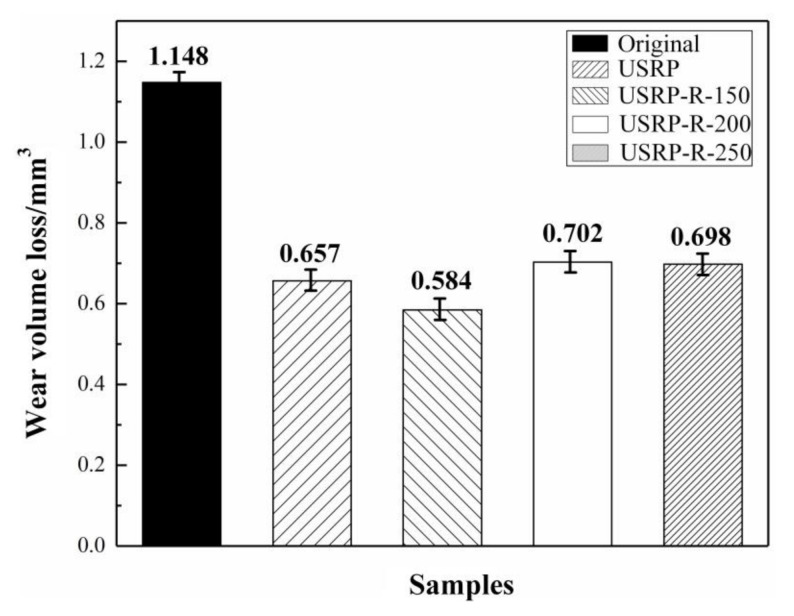
The wear volume losses of the original sample, USRP sample and USRP-R samples.

Figure 12 shows the wear volume losses of the original, USRP and USRP-R samples under a 10 N load over a sliding time of 30 min. The wear volume loss is measured by the cross-sectional area and length of the wear scar of the sample. As shown, the wear volume losses of the original sample, USRP sample, USRP-R-150 sample, USRP-R-200 sample and USRP-R-250 sample are 1.148 mm^3^, 0.657 mm^3^, 0.584 mm^3^, 0.702 mm^3^ and 0.698 mm^3^, respectively. Obviously, the wear volume loss of the original sample is larger than that of the USRP sample and USRP-R samples. According to the Archard equation [37], wear volume loss is usually inversely proportional to the hardness of the material, that is, the higher the hardness, the smaller the wear volume loss. However, the wear volume loss of the USRP-R-150 sample is smaller than that of the USRP sample, while the surface microhardness of the USRP-R-150 sample is lower than that of the USRP sample. The result reveals that the wear resistance of Mg alloy is not only related to its microhardness, but also to its toughness. Somekawa et al. [38] found that a suitable annealing treatment was helpful to the release of internal stress and the improvement of toughness. The improvement of toughness of the USRP-R sample is attributed to the transformation of the dislocation cells into fine grains. In addition, when the recovery temperatures are 200 °C and 250 °C, microhardness that is too low results in poor wear resistance of AZ91D Mg alloy.

**Figure 13:**
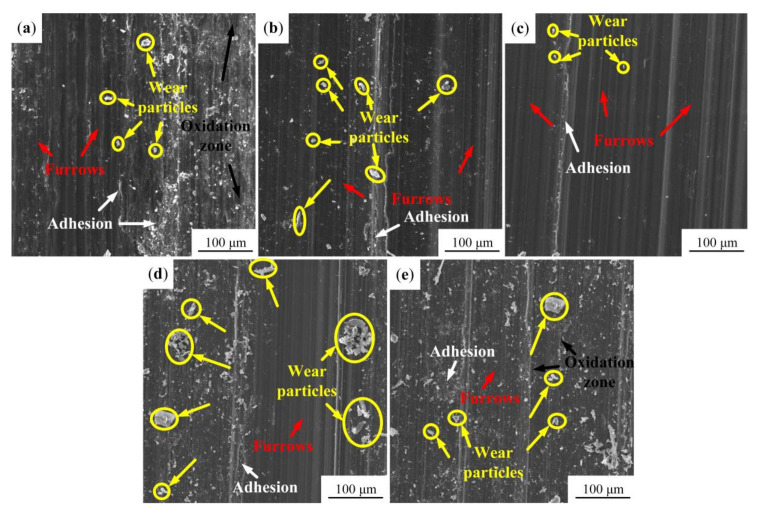
The worn surface morphologies of samples: (**a**) the original sample; (**b**) the USRP sample; (**c**) the USRP-R-150 sample; (**d**) the USRP-R-200 sample; (**e**) the USRP-R-250 sample.

The worn surface morphologies of different samples are shown in Figure 13. As shown in Figure 13a, a large number of wear particles and parallel furrows can be observed on the worn surface of the original sample, indicating that there is severe abrasive wear under the dry sliding friction condition. Black oxidation zones appear on the worn surface, presenting as typical oxidation wear. This is because Mg alloy matrix is easily oxidized in air, and it is promoted by heat generated by dry sliding friction. At the same time, adhesive particles can be observed on the worn surface. Therefore, the wear mechanism of the original sample is severe abrasive wear, oxidation wear and adhesive wear. Figure 13b gives the wear morphology of the USRP sample. The shallow and parallel furrows are distributed along the sliding direction on the worn surface, which are produced by wear particles (as marked). In addition, slight adhesion can be found on the worn surface. As a result, the wear mechanism of the USRP sample is abrasive wear and adhesive wear. The worn surface of the USRP-R-150 sample shown in Figure 13c is relatively smooth. Only a small amount of wear particles are distributed on the worn surface and the furrows are narrow and shallow. This demonstrates that the wear resistance of the USRP sample after a recovery treatment at 150 °C is greatly improved. Moreover, a slight adhesive phenomenon is also observed on the worn surface. Therefore, the wear mechanism of the USRP-R-150 sample is abrasive wear and adhesive wear. Figure 13d shows the wear morphology of the USRP-R-200 sample. A large number of large wear particles are distributed on the worn surface, illustrating that the abrasive wear on the surface of the USRP-R-200 sample is serious. Meanwhile, adhesion and spalling appeared on the worn surface, indicating that adhesive wear has occurred. Consequently, the wear mechanism of the USRP-R-200 sample is abrasive wear and adhesive wear. The worn surface morphology of the USRP-R-250 sample is similar to that of the USRP-R-200 sample (Figure 13e). Many wear particles and adhesive phenomenon are found on the worn surface of the USRP-R-250 sample. In addition, the heat generated during the sliding process leads to the formation of oxide film on the worn surface of the sample, resulting in oxidation wear, as shown by the black arrow in the figure. Therefore, the wear mechanism of the USRP-R-250 sample is abrasive wear, adhesive wear and oxidation wear.

**Figure 14:**
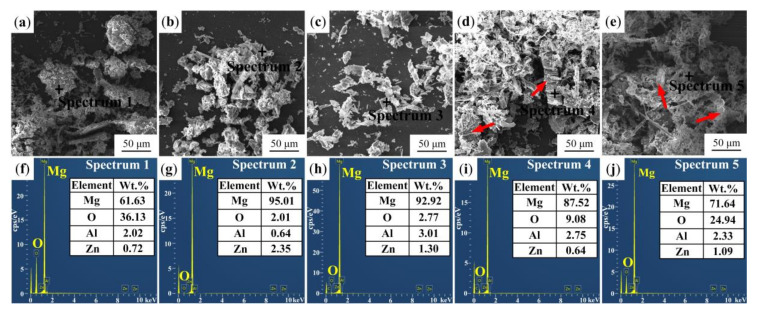
The morphologies and point scanning results of wear particles of the different samples: (**a**,**f**) the original sample; (**b**,**g**) the USRP sample; (**c**,**h**) the USRP-R-150 sample; (**d**,**i**) the USRP-R-200 sample; (**e**,**j**) the USRP-R-250 sample.

The investigation of wear particles is also an effective way to analyze the wear behavior of samples. The SEM images and main elements of wear particles with different morphologies are shown in Figure 14. The wear particles of the original sample shown in Figure 14a are large and flake-shaped, indicating that the worn surface of the original sample is seriously exfoliated. The high content of oxygen is found in wear particles by point scanning, which illustrates that the worn surface is seriously oxidized during the dry sliding friction (Figure 14f). Figure 14b,c show the morphologies of wear particles of the USRP sample and USRP-R-150 sample, respectively. The wear particle sizes of the two samples are both smaller than that of the original sample. Meanwhile, compared with the USRP-R-150 sample, the wear particle size of the USRP sample is larger, exhibiting that the wear of USRP sample is more serious. Hwang et al. [39] found that in the process of friction and wear, the larger the wear particles, the higher the FC of the sample. In addition, the contents of oxygen in wear particles of the USRP sample and USRP-R-150 sample are both very low, indicating that they are not seriously oxidized during the friction and wear test. This can be attributed to the lower FCs of the two samples, resulting in lower frictional heat, compared with the original sample. As shown in Figure 14d,e, the wear particles of the USRP-R-200 sample and USRP-R-250 sample are mainly ribbon-shaped (shown by arrowheads), which is a typical characteristic of abrasive wear. Additionally, the content of oxygen of the USRP-R-250 sample is higher than that of the USRP-R-200 sample, which is consistent with the worn morphology of the USRP-R-250 sample shown in Figure 14e.

## 4. Conclusions

In this study, the effects of USRP and subsequent recovery treatment on the wear resistance of AZ91D Mg alloy were investigated. The main research conclusions are summarized as follows:

(1) The surface grains of the USRP sample are refined to nanocrystallines with sizes ranging from 50 nm to 70 nm, and the thickness of the severe plastic deformation layer is about 190 μm. The grain refinement mechanism of AZ91D Mg alloy after USRP is twinning, dislocation slip and dynamic recrystallization.
(2) Compared with the original sample, the surface roughness of the USRP sample decreases significantly from 1.283 μm to 0.118 μm. In addition, the microhardness of the USRP sample decreases along the depth direction of the cross section. The maximum microhardness of the top surface for the USRP sample reaches 102.3 HV, which is about 1.7 times the original sample.
(3) The recovery treatment has effects on the microstructure and mechanical properties of AZ91D Mg alloy treated by USRP. With the increase of temperature, the thickness of the deformation layer decreases and the grains expand. The microhardness of all the USRP-R samples is lower than that of the USRP sample. The microhardness of the top surface of the USRP-R samples is 90.68 HV, 79.29 HV and 75.06 HV at the recovery temperature of 150 °C, 200 °C and 250 °C, respectively.
(4) The wear resistance of the USRP sample is much better than that of the original sample, mainly due to the high microhardness. Moreover, the wear resistance of the USRP sample is further improved after a recovery treatment at 150 °C, which is attributed to the improvement of toughness. However, when the recovery temperatures are 200 °C and 250 °C, the wear resistance of the samples is poor due to low microhardness. The wear resistance of AZ91D Mg alloy depends not only on hardness but also on toughness.

## Figures and Tables

**Table 1:** The chemical composition of AZ91D Mg alloy (wt.%).

Al	Zn	Mg	Si	Fe	Ni	Cu	Mg
8.72	0.51	0.31	0.04	0.003	0.001	0.003	Bal.

**Table 2:** The parameters of ultrasonic surface rolling processing (USRP).

Current/A	Frequency/KHz	Amplitude/μm	Spindle Speed/r·min^−1^	Load/N	Feed Rate/mm·r^−1^
0.7	20	7.5	80	240	0.1
